# Identification, characterization, antimicrobial activity and biocontrol potential of four endophytic fungi isolated from Amazonian plants

**DOI:** 10.1038/s41598-025-26865-6

**Published:** 2025-11-10

**Authors:** Sonia Mendieta-Brito, Mahmoud Sayed, Faqiha Ali Hamza, Eunjung Son, Dong-Seon Kim, Giovanna Plata, Marcelo Dávila, Sang-Hyun Pyo

**Affiliations:** 1 Biotechnology and Applied Microbiology, Department of Process & Life Science Engineering, Faculty of Engineering, Lund University, Lund, SE-22100 Sweden; 2 Centro de Tecnología Agroindustrial, Universidad Mayor de San Simón, Cochabamba, Bolivia; 3 Department of Botany and Microbiology, South Valley University, Qena, 83523 Egypt; 4 KM Science Research Division, Korea Institute of Oriental Medicine, 1672 Yuseong-daero, Yuseong-gu, Daejeon, 34054 Republic of Korea; 5 Fundación “Promoción e Investigación de Productos Andinos” (PROINPA), Zona El Paso Quillacollo, Cochabamba, Bolivia

**Keywords:** Endophytic fungi, Molecular identification and characterization, Antimicrobial activity, Antagonistic and biocontrol assay, Amazonian biodiversity, Microbiology, Fungi, Antimicrobials

## Abstract

Endophytic fungi, which reside within plants without causing disease, are recognized for their ability to produce bioactive metabolites with antibacterial, antifungal, and antioxidant properties, as well as their role in enhancing plant defense mechanisms. Due to these valuable traits, endophytic fungi have attracted significant attention in biotechnology and microbiology. The four endophytic fungal strains were isolated from the leaves of four Amazonian plant species—*Piper heterophyllum* Ruiz & Pav. (Paichané negro), *Peperomia* sp., *Faramea multiflora* A. Rich. ex DC.(Yuracaré), and *Dictyoloma vandellianum* A. Juss. (Sombrerillo). Molecular identification via 18 S rDNA sequencing and NCBI-BLAST analysis, as well as morphological characterization, were carried out for the isolates. Ethyl acetate extracts were obtained from both the growth medium and the fungal biomass. Thin-layer chromatography (TLC) combined with various staining techniques was used to identify the main groups of chemical compounds present in the extracts. The extracts were then assessed for antibacterial activity through a minimum inhibitory concentration (MIC) assay. The antagonistic potential of four endophytic fungi was evaluated through confrontation with phytopathogenic fungi using the dual culture plate assay. The results from molecular and morphological identification revealed two *Aspergillus* strains (SMB-18 and SMB-22), one *Fusarium* strain (SMB-20), and one *Alternaria* strain (SMB-28). Chemical profiling revealed a diverse composition, including carotenoids, terpenes, flavonoids, and phenolic compounds. The MIC assay demonstrated strong antibacterial activity against Gram-positive bacteria (*Staphylococcus aureus*, *Enterococcus faecalis*, and *Propionibacterium acnes*), with MIC values ranging from 15.6 to 500 µg/mL. Additionally, antagonistic and biocontrol assays using dual-culture tests showed strong antifungal activity. Strains SMB-18, SMB-20, and SMB-22 effectively inhibited *Helminthosporium* sp. (58–80%), *Fusarium oxysporum* (37–50%), and *Fusarium solani* (51–57%), the well-known phytopathogenic fungi that affect potato crops. These findings highlight the potential of Amazonian endophytic fungi as sources of bioactive metabolites with promising applications in agriculture, medicine, and biotechnology, reinforcing the importance of biodiversity in bioprospecting.

## Introduction

Endophytic fungi reside within healthy plant tissues and play a crucial role in the plant’s microecosystem. Their population dynamics are influenced by factors such as the genetic makeup, age, and environmental conditions of the host plant^1,2^. These fungi establish symbiotic relationships with their hosts, producing important bioactive compounds such as alkaloids, diterpenes, flavonoids, and isoflavonoids. These compounds contribute to plant growth, enhance resilience, and strengthen defense mechanisms against environmental stressors, pests, and diseases while also promoting the accumulation of secondary metabolites^3,4^.

Many bioactive compounds produced by endophytic fungi are chemically identical or similar to those synthesized by their host plants^5^. Examples include Taxol^6,7^, capsaicin^8^, and piperine^9^. The in vitro cultivation of endophytes capable of producing these metabolites presents a sustainable alternative to harvesting plants from natural populations, reducing environmental impact, and ensuring consistent production irrespective of climatic conditions^10,11^. Therefore, endophytic fungi have gained significant attention in biotechnology and industry for their applications as biocontrol agents, antimicrobials, antitumor agents, antioxidants, antidiabetics, antibiotics, and insecticides^12,13^.

Regarding antibacterial activity, alkaloids, terpenoids, polyketides, lactones, and phenolic substances produced by endophytic fungi target the bacterial cell walls, alter membrane permeability, or inhibit essential enzymes. For example, an endophyte isolated from *Azadirachta indica* produces alkaloids and lactones that inhibit *S. aureus* and *B. subtilis*^14^. While Gram-negative bacteria are generally more resistant due to their outer membrane, fungi like *Colletotrichum* produce compounds that penetrate this barrier, effectively inhibiting *E. coli* and *P. aeruginosa*^15^. Some fungi, such as *Beauveria bassiana*, exhibit broad-spectrum activity, producing compounds like beauvericin that inhibit both Gram-positive and Gram-negative bacteria^5^.

Endophytic fungi are also a rich source of antifungal agents. Their metabolites demonstrate potent activity against human and plant fungal pathogens^16^. These mechanisms include the production of primary metabolites, secondary metabolites, and volatile organic compounds (VOCs) that limit pathogen growth. For instance, the fungus *Trichoderma* spp. produces peptaibols and trichodermolides, which effectively inhibit phytopathogenic fungi of the genera *Fusarium* and *Botrytis*^5^. Similarly, the fungus *Colletotrichum* exhibits antifungal activity against *Phytophthora infestans*, the causal agent of late blight. Other genera, such as *Aspergillus* and *Penicillium*, are well-known for their production of secondary metabolites, including alkaloids and volatile compounds, which possess strong antifungal properties. Additionally, *Phomopsis* spp., an endophytic fungus isolated from medicinal plants, demonstrates antifungal activity against human pathogens *Candida albicans* and *Aspergillus fumigatus*^17,18^.

Criteria include plants with ethnobotanical significance, which are known to have traditional medicinal uses, and those growing in biodiversity hotspots. Tropical rainforests, with their intense competition and evolutionary pressures, offer a high likelihood of yielding novel molecular structures and bioactive compounds^14,19^.

The use of biological control for managing plant pests has recently gained significant attention. Many biological control agents, particularly endophytic fungi, are naturally associated with crops and play a crucial role in suppressing harmful pathogens^20^. Fungal pathogens can severely impact plant physiology, triggering host defense mechanisms in response to infection. In Bolivia, potato cultivation is essential for food security but faces significant threats from fungal diseases such as *Phytophthora infestans* (late blight), *Fusarium solani*, and *Alternaria solani*. These pathogens can cause yield losses of 30–50%, depending on management practices. National efforts, led by organizations like the PROINPA Foundation, focus on identifying these diseases and developing integrated control strategies, including resistant crop varieties and improved cultural practices^21^.

The plant species used in this study were selected and collected for their traditional medicinal uses (Table 1). In particular, species from the *Piper* genus have long been used to treat a range of conditions, including urological problems, skin, liver, and stomach disorders, and to support wound healing. They are also known for their antipyretic (fever-reducing) and anti-inflammatory effects. Additionally, *Piper* species show promise as natural antioxidants and antimicrobial agents for food preservation. Research indicates that their phytochemicals and essential oils, especially phenolic compounds, exhibit strong antioxidant activity, sometimes even outperforming synthetic alternatives. These compounds, along with monoterpenes and sesquiterpenes, also contribute to their antibacterial and antifungal properties against human pathogens^22^.

**Table 1:** Medicinal plants of the Valle Del sacta in Cochabamba, Bolivia.

Scientific name of the plant	Common name	Part of the plant used	Collection location (This study)	Traditional use	Reference
*Piper heterophyllum* Ruiz & Pav.	Paichané negro Verdillo	Leaf	Valle del Sacta, Cochabamba, 17°06’06"S and 64°46’54"W, 240 m.a.s.l	Runny nose or ear, fright, muscle pain, knee rheumatism, stomach pain, scabies	Botanical expert ^22^
*Peperomia* sp.		Leaf	Valle del Sacta, Cochabamba 17°06’06"S and 64°46’54"W, 240 m.a.s.l.	Anti-inflammatory and pain	Botanical expert ^23^
*Faramea multiflora* A. Rich. ex DC.	Yuracaré	Leaf	Valle del Sacta, Cochabamba 17°06’00"S and 64°46’54"W, 240 m.a.s.l.	Stomach pain and diarrhea	Botanical expert ^24^
*Dictyoloma vandellianum* A. Juss	Sombrerillo	Leaf	Valle del Sacta, Cochabamba 17°06’31"S and 64°46’40"W, 235 m.a.s.l.	Joint inflammation, pain	Botanical expert ^25^

Plants from the *Peperomia* genus (within the Piperaceae family) are also recognized for their medicinal potential, with effects such as antihypertensive, anti-inflammatory, pain-relieving (antinociceptive), antiplasmodial, and osteogenic activity. Among them, *Peperomia pellucida* stands out for its strong antioxidant activity, largely due to its high levels of terpenoids, phenolic compounds, and flavonoids^23^.

Similarly, the species of the *Faramea* genus have been traditionally used to treat wounds, fevers, and digestive issues, thanks to their anti-inflammatory, analgesic, and antimicrobial properties. These effects are mainly attributed to the presence of bioactive compounds like alkaloids, flavonoids, and terpenoids, common in members of the Rubiaceae family. Some research suggests that related species possess antimicrobial activity, which may also be true for *Faramea multiflora*^24^.

Finally, *Dictyoloma vandellianum* A. Juss., from the Rutaceae family, has been traditionally used to relieve inflammation and pain. Its extracts contain bioactive compounds such as alkaloids, coumarins, and terpenoids that have demonstrated antimicrobial effects against various bacteria and fungi. In addition, its flavonoids and phenolic compounds contribute significantly to its antioxidant capacity^25^. In traditional Bolivian medicine, the leaves are widely used, especially in infusions, with the understanding that these are synthesis sites for a variety of active compounds^26,27^.

In this study, four medicinal plants from the Amazon region of Cochabamba, Bolivia, were collected. Four endophytic fungal species were isolated from their leaves and identified through 18S ribosomal DNA sequencing, phylogenetic analysis using NCBI-BLAST, and morphological characterization. The crude extracts from these fungi were tested for antibacterial activity using a minimum inhibitory concentration (MIC) assay, demonstrating strong efficacy against Gram-positive bacteria. Additionally, their antagonistic potential was assessed through confrontation with major phytopathogenic fungi affecting potato crops using the dual culture plate assay, revealing high antifungal activity. These findings highlight endophytic fungi as promising sources of biologically active secondary metabolites with potential applications in medicine, pharmaceuticals, agriculture, and environmental biotechnology.

## Methods

### Materials

Media used for the cultivation of Endophytic fungi and phytopathogenic fungi were Potato Dextrose Agar (PDA) and Potato Dextrose Broth (PDB), prepared following standard protocols^28^. For bacterial cultures, Mueller-Hinton Agar (MH), Trypticasein Soy Broth (TSB), and Trypticasein Soy Agar (TSA) (Oxoid, UK) were used. DNA extraction was performed using the Quick-DNA Fungal/Bacterial Miniprep Kit (Zymo Research, Irvine, CA, USA). PCR products were purified with the GeneJET PCR Purification Kit (Thermo Scientific, Waltham, MA, USA). For polymerase chain reaction (PCR) amplification, 18S rDNA-specific primers (EF4f and EF3r) from Integrated DNA Technologies (IDT, Coralville, IA, USA) were utilized. Chemical extraction and separation processes involved ethyl acetate, ethanol, sodium hypochlorite, *p*-anisaldehyde, ferric chloride, and aluminum chloride, which were purchased from Sigma-Aldrich (St. Louis, MO, USA).

### Isolation of endophytic fungi from Amazonian plants

Four Amazonian plant species were collected in Valle del Sacta, located within Carrasco Province, Cochabamba, Bolivia. The collection took place on the Universidad Mayor de San Simón property with the necessary permits granted by the authorities and in compliance with legal regulations (Table 1).

Plant identification was conducted by a professional expert using taxonomic keys for Amazonian and regional flora. Verification was carried out at the “Martín Cárdenas” National Forest Herbarium (BOLV), where a voucher specimen of each species was deposited. The collected plant material, consisting of small branches with leaves, was stored in refrigerated containers at 4 °C for transport to the laboratory^19^.

Endophytic fungi were isolated from healthy leaves. To do this, the leaf surface was disinfected by sequential washing with running water, 70% ethanol (2 min), 1% sodium hypochlorite (1 min), and sterilized water (2 min)^14,19,29–32^. The plant material was subsequently cut into fragments of approximately 5 mm² and plated on Petri dishes with PDA medium supplemented with chloramphenicol (100 µg/mL), using 8–10 fragments per plate. The plates were incubated at 22 °C for 15 days. The emerging fungal mycelium was transferred to plates with fresh medium for purification, obtaining axenic strains that were stored for subsequent characterization. For long-term conservation, the purified strains were suspended in PDB with 10% glycerol as a cryoprotectant, placed in sterile cryovials, and stored at −20 °C, to preserve their viability and ensure their stability for future studies^33,34^. All isolated fungal strains were deposited in the Microbiology Laboratory of the Centro de Tecnología Agroindustrial (CTA) at Universidad Mayor de San Simón, Cochabamba, Bolivia.

### Identification of endophytic fungi

Four endophytic fungal strains were isolated from the leaves of four medicinal plant species, including *Piper heterophyllum* Ruiz & Pav. (Paichané negro), *Peperomia* sp., *Faramea multiflora* A. Rich. ex DC.(Yuracaré), and *Dictyoloma vandellianum* A. Juss. (Sombrerillo) (Table 1). Identification of endophytic isolates was carried out using both classical taxonomic methods based on morphological characteristics and molecular techniques.

For the macromorphological characteristics, after seven days of culturing at 30 °C on PDA in 10 × 100 mm Petri dishes, macroscopic vegetative traits were examined. These included colony characteristics such as color, texture, topography, diffuse pigmentation, and colony borders. Microscopic features, including hyphal structures and reproductive elements, were assessed using the microculture technique on PDA and yeast extract sucrose (YES) media for 7–10 days. Fungal samples were stained with Lactophenol blue and observed under an optical microscope (LRI - Olympus-100×/0.65, Tokyo, Japan). Observations were then compared with established taxonomic keys^35–37^.

The molecular identification was performed by amplifying the 18S rDNA gene using specific PCR primers listed in Table 2. DNA template was obtained through the collection of 50–100 mg wet weight of fungal samples from fresh cultures grown on PDA plates at 28–30 °C for 7–15 days. Thereafter, genomic DNA extraction was performed using the Quick-DNA Fungal/Bacterial Miniprep Kit (Zymo Research, Irvine, CA, USA), following the manufacturer’s protocol^38^and stored at −20 °C in 100 µL of DNA elution buffer.

For PCR amplification, EF4f and EF3r primers (Table 2) were used to target the 18S rDNA gene from fungal DNA (SMB-18, SMB-20, SMB-22, and SMB-28). PCR reactions were performed in a T100 Thermal Cycler (Bio-Rad Laboratories Inc., Hercules, CA, USA) under the conditions outlined in Table 2. Following PCR, the products were purified and sequenced, and phylogenetic trees were constructed using the methodology described by Mendieta-Brito et al.^32^ for the identification of the fungal strains. In all obtained phylogenetic trees for the four fungal species, the scale bar indicates nucleotide substitutions per site, using the neighbor-joining method. The number of nodes indicates the bootstrap values of 1000 replicates. The model used was Jukes-Cantor (JC).

**Table 2:** Molecular markers and their PCR primers and programs used.

Loci	PCR primers	Sequence (5´- 3´)	PCR cycles			Ref.
			Denaturation	Annealing	Polymerization	
TEF-α	F: EF4f R: Fung5r	GGA AGG GGA TGT ATT TAT TAG GTA AAA GTC CTG GTT	94 °C: 2 min, 35 cycles 94 °C: 30 s	53 °C: 30 s	72 °C: 1 min 72 °C: 1 min 4 °C: ∞	^35,58^
TEF1-α	F: EF4f R: EF3r	GGA AGG GGA TGT ATT TAT TAG TCCTCTAAATGACCAGTTTG	94 °C: 2 min, 35 cycles 94 °C: 30 s	53 °C: 30 s	72 °C: 1 min 72 °C: 1 min 4 °C: ∞	^59,60^

### Chemical profile of crude extract from endophytic fungi

For the preparation of crude extracts, five 5 × 5 mm mycelium fragments from each endophytic fungal strain (SMB-18, SMB-20, SMB-22, and SMB-28) were excised from PDA plates and transferred to 500 mL Erlenmeyer flasks containing 200 mL of PDB. The cultures were incubated in a shaker incubator for 15 days at 30 °C and 200 rpm.

After incubation, the fungal biomass was harvested using vacuum filtration through a Büchner funnel fitted with standard filter paper. For the liquid-medium fraction, a liquid–liquid extraction technique was employed using ethyl acetate with a volume ratio of 1:1, and three extractions were performed. The remaining mycelium portion underwent maceration with twice its volume of ethyl acetate for 24 h. Subsequently, the extracted fractions from both mycelium and broth were combined, and the solvent was evaporated using a rotary evaporator under vacuum, with the water bath set at 30℃. Thin-layer chromatography (TLC) was employed to analyze the chemical profile of these crude extracts. TLC plates were developed using suitable eluents hexane: ethyl acetate (1:1); and developer solutions to identify and compare the compounds present in the extracts. The Retention factor (Rf) of each compound was calculated as the ratio of the distance migrated by the compound to the distance migrated by the eluent front, according to the methodology described by Mendieta-Brito et al.^32^.

### Antibacterial test: determination of minimum inhibitory concentration (MIC)

The antimicrobial activity of the crude fungal extracts was assessed by determining the minimum inhibitory concentration (MIC) using a microdilution method, as described by the Clinical and Laboratory Standards Institute^40,41^, with slight modifications. Since in pre-test on disc diffusion assay, the sample dried from aquouse phase wasn’t shown the activity against to gram negative and positive mircroorganisms, ethylacetate extracts were evaluated on MIC. The MIC was evaluated against the following five bacterial strains that were obtained from the Korean culture collections (KCTC): *Staphylococcus aureus* (KCTC 3881, Gram-positive), *Escherichia coli* (KCTC 1039, Gram-negative), *Pseudomonas aeruginosa* (KCTC 1637, Gram-negative), *Enterococcus faecalis* (KCTC 2011, Gram-positive), and *Propionibacterium acnes* (KCTC 6919, Gram-positive).

Bacterial cultures were grown under specific conditions: *E. coli*, *S. aureus*,* and P. aeruginosa* were incubated aerobically at 37 °C in Nutrient Broth (MBcell, South Korea), *E. faecalis* and *P. acnes* were cultured at 37 °C under facultative anaerobic conditions in Brain Heart Infusion (BHI) Broth (MBcell, South Korea). The bacterial suspensions were diluted until the absorbance at 660 nm reached 0.03, corresponding to a concentration of approximately 1–2 × 10^7^ CFU/mL.

The test was performed in a sterilized 96-well microplate (Falcon, Dublin, OH, USA), with 1 mL of fungal extract serially diluted in each well. The initial concentration of the extracts was adjusted to 1000 µg/mL, followed by serial twofold dilutions to 500, 250, 125, 62.5, 31.3, and 15.6 µg/mL. To each well, 10 µL of bacterial culture medium was added. The microplates were incubated for 12 h at 37 °C with shaking (150 rpm). The bacterial growth inhibition was quantified by measuring the optical density at 620 nm (OD620) using a spectrophotometer (BIO-RAD Laboratories Inc., USA).

Ampicillin was used as a positive control, with known MIC values: 1.25 µg/mL for *E. coli* and *E. faecalis*, and a range of 20–0.625 µg/mL for *S. aureus* (MIC90 not determined). The MIC ranges of 1.0–0.05 µg/mL for *P. aeruginosa* and *P. acnes* were also determined based on ampicillin’s known activity.

### Antifungal test: direct confrontation—dual culture plate assay

Phytopathogenic fungi, including *Helminthosporium* sp., *Fusarium oxysporum*, and *Fusarium solani*, which are of agronomic importance to potato crops, were obtained from the microbiology and phytopathology laboratories of the PROINPA Foundation.

The antagonistic activity of the endophytic fungi, *Aspergillus* sp. SMB-18, *Fusarium* sp. SMB-20, *Aspergillus* sp. SMB-22, and *Alternaria* sp. SMB-28 was evaluated against the phytopathogenic fungi, using a dual culture assay. In the assay, the inoculation of the endophytic fungi and the pathogen in a paired fashion on the surface of PDA or YES plates was done. Where each fungus (endophyte and pathogen) was inoculated 2.1 cm apart from the perimeter of the Petri dish^42^.

The inoculation was performed in triplicate. Also, a control plate containing only the pathogen was included to monitor its uninhibited growth. All plates were incubated in a New Brunswick Innova 4200 Incubator (Edison, NJ, USA), under standardized conditions (natural light/dark cycles, humidity 30–40%, temperature 23–25 °C) to support optimal microorganism growth.

Pathogen growth was measured at regular intervals (7, 10, 14, and 18 days), depending on the maximum growth observed in the control plates. The pathogen inhibition was calculated by comparing the radial growth of the pathogen on treated plates (facing the endophyte) with its growth on control plates^43^. The percentage of mycelial growth inhibition was determined using the following formula:

$$\:Inhibition\:Percent\:\left(I\right)\:=\:\left[\right(R1\:-\:R2)\:\div\:R1]\:\times\:\:100,$$

where R1 = radial growth of the pathogen in control and R2 = radial growth of the pathogen in treatment^44^.

Additionally, the interaction between the endophytic fungi and phytopathogens was categorized according to established classification systems (Table 3) based on the observed inhibitory effects against the growth of the pathogens.

**Table 3:** Types of the interaction categories between endophytic and pathogenic fungi.

Categories	Description	Reference
Type A	Mutual intermingling of mycelial growth in which both fungi grow into each other without any macroscopic interaction	^44^
Type B	Mutual inhibition on contact or < 2 mm inhibition zone is observed between the colonies of two fungi	
Type C	Significant inhibition of one species on contact, inhibited species continuously grow at a significantly reduced rate, whereas the inhibitor species grow at a slightly reduced rate	
Type D	More than 2 mm inhibition zone observed between the growth of two fungi.	
Type E	Inhibition of one species on contact, and the inhibitor species continue to grow with a reduced rate through inhibited colony	
Type F	Growth inhibition of one species on contact or at a distance, inhibitor species continue to grow at unchanged rate through or over the inhibited species	

## Results

### Isolation and molecular identification of endophytic fungi

**Fig. 1:**
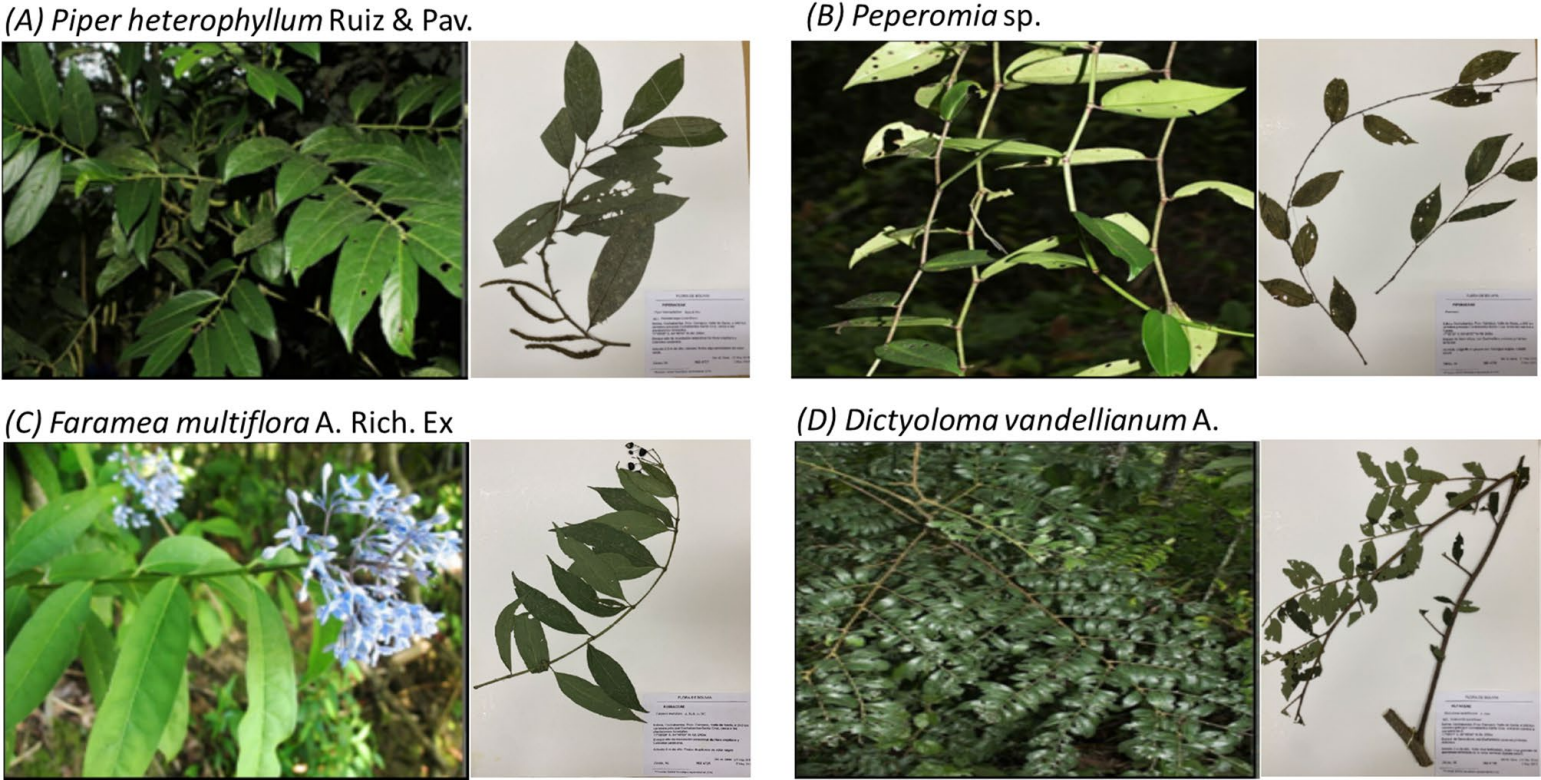
Collection of plant species in the Amazon region called Valle del Sacta at an altitude of 240 m above sea level, Cochabamba-Bolivia (A) *Piper heterophyllum* Ruiz & Pav. (17°06’06"S and 64°46’54"W) (B) *Peperomia* sp. (17°06’06"S and 64°46’54"W) (C) *Faramea multiflora* A. Rich. Ex DC. (17°06’00"S and 64°46’54"W) and (D) *Dictyoloma vandellianum* A. Juss. (17°06’31"S and 64°46’40"W), to the right of the photo of each plant is the sample assembled in a Micro-herbarium.

Figure 1 presents an image of the collected host plants from the Amazon region of Bolivia. These plants were identified by Mgr. Modesto Zarate, an associate researcher at the Herbarium, and their identification was corroborated by reference to the “Martín Cárdenas” National Forest Herbarium (BOLV).

The species, known in their regions of origin for their medicinal uses (Table 1), were identified as *Piper heterophyllum* Ruiz & Pav., *Peperomia* sp., *Faramea multiflora* A. Rich. ex DC., and *Dictyoloma vandellianum* A. Juss. Voucher specimens are available under the following accession numbers: MZ6727, MZ6732, MZ6725, and MZ7738, respectively.

**Fig. 2:**
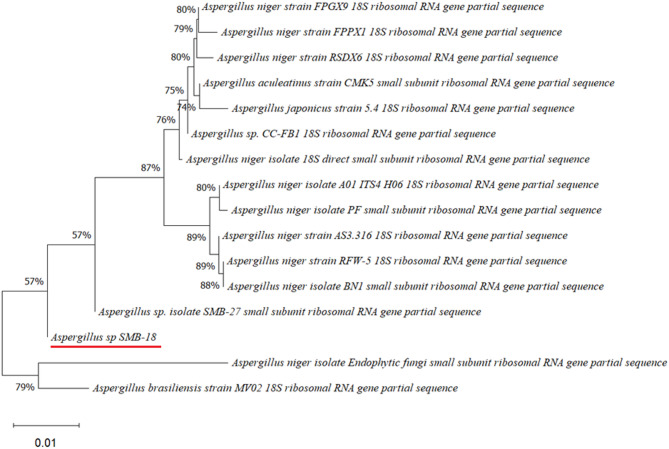
Phylogenetic tree based on the partial sequence of a small-subunit ribosomal RNA gene of endophytic fungus *Aspergillus* sp. SMB-18 (accession no. PQ490368) obtained with EF4f/Fung5r, showing its relationship via neighbor-joining with other closely related taxa from NCBI GenBank.

**Fig. 3:**
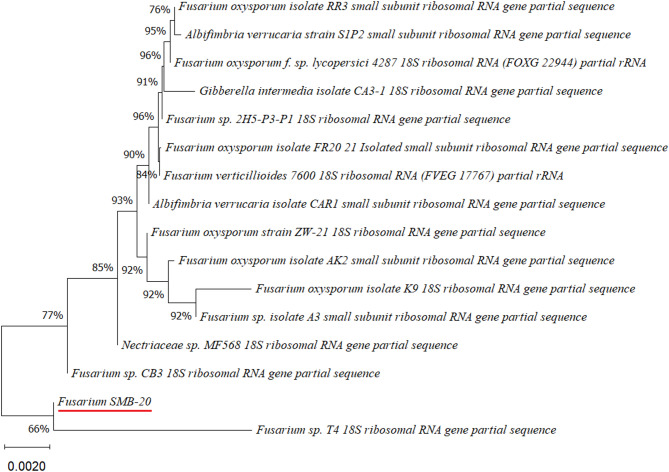
Phylogenetic tree based on the partial sequence of a small-subunit ribosomal RNA gene of endophytic fungus *Fusarium* sp. SMB-20 (accession no. PQ483103) obtained with EF4f/EF3r, showing its relationship via neighbor-joining with other closely related taxa from NCBI GenBank.

**Fig. 4:**
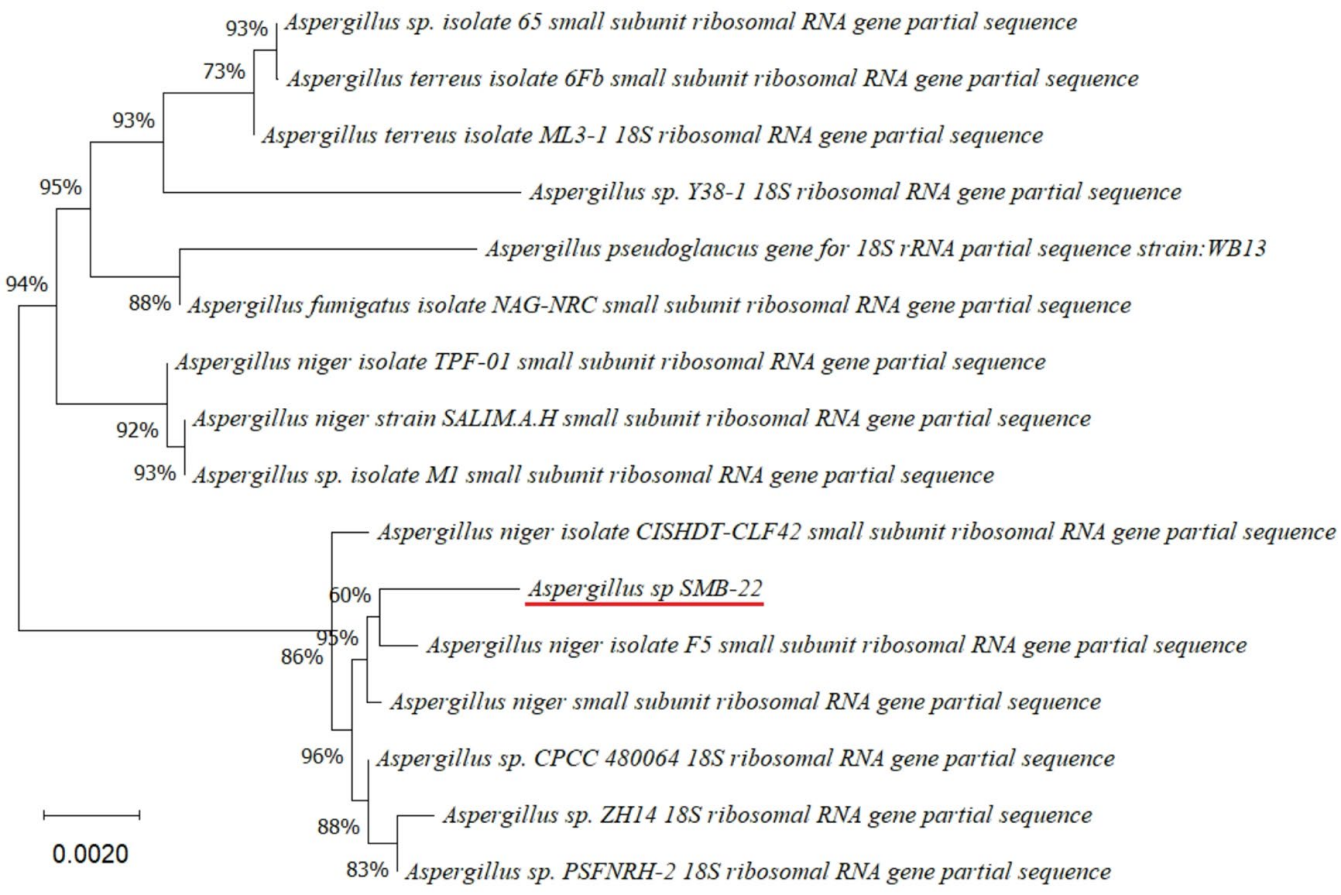
Phylogenetic tree based on the partial sequence of a small-subunit ribosomal RNA gene of endophytic fungus *Aspergillus* sp. SMB-22 (accession no. PQ483115) obtained with EF4f/EF3r, showing its relationship via neighbor-joining with other closely related taxa from NCBI GenBank.

**Fig. 5:**
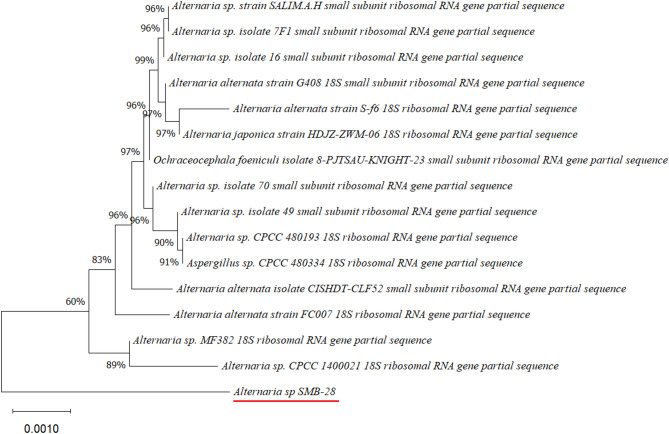
Phylogenetic tree based on the partial sequence of a small-subunit ribosomal RNA gene of endophytic fungus *Alternaria* sp. SMB-28 (accession no. PQ483124) was obtained with EF4f/EF3r, showing its relationship via neighbor-joining with other closely related taxa from NCBI GenBank.

**Fig. 6:**
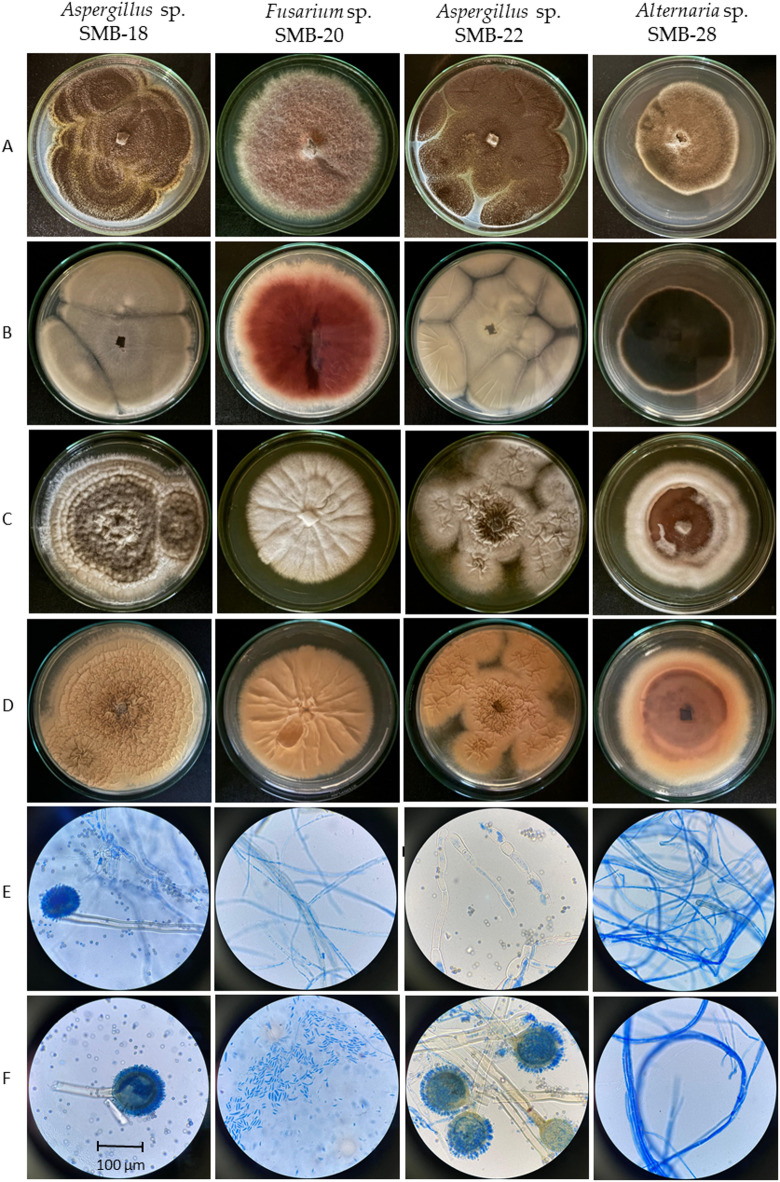
Macro- and micromorphological characteristics of endophytic fungi: Colonies on PDA upper (A) and reverse side (B) after 7 days at 30 °C. Colonies on YES upper (C) and reverse side (D) after 7 days at 30 °C. Generative hyphae and mycelium (E) and conidiophores and conidia (F). Samples stained with Lactophenol blue, observed under an LRI-Olympus microscope at 100×/0.65 magnification, and a scale of 1:200.

Four endophytic fungi were successfully isolated from the leaves of the four medicinal plants (Figs. 2, 3, 4, 5 and 6). The fungal isolates were subjected to both morphological and molecular identification processes to determine their taxonomic classification (Figs. 2, 3, 4,5 and 6, and Tables 4 and 5).

**Table 4:** GenBank accession numbers for the isolated endophytic fungi.

Specie	Source	GenBank Accession Number
*Aspergillus* sp. SMB-18	Leaf	PQ490368
*Fusarium* sp. SMB-20	Leaf	PQ483103
*Aspergillus* sp. SMB-22	Leaf	PQ483115
*Alternaria* sp. SMB-28	Leaf	PQ483124

**Table 5:** Cultural and morphological characteristics of 4 endophytic fungal isolates from Amazonian Bolivian plants.

	Macroscopic and microscopic characteristics								
Endophytic fungi strains	Colonies (PDA)	Colonies (YES)		Size colonies		Hyphae	Conidiophores and conidia		References
*Aspergillus niger*	The growth is initially white but changes to black after a few days producing conidial spores. The edges of the colonies appear pale yellow, producing radial fissures.			Cottony appearance, initially white to yellow and then turning black. The reverse rough yellow on PDA. In YES, the color is and more orange on the reverse.		Filamentous fungus that forms filamentous hyphae that make them look like small plants.	The conidial heads appear radial, they are smooth and hyaline. The conidiophore becomes dark at the apex and terminating in a globose vesicle which is 30–75 μm in diameter. Produce conidia brown colored and have a diameter of 4–5 μm.		^35,36^
*Aspergillus* sp, SMB-18	Cottony appearance, initially white to yellow in color and then turning black. With formation of many black spores. The reverse is pale yellow and rough	Brown with white concentric edges. Orange light rough appearance on the reverse		Colony growth reached 90 mm after the seventh day on PDA at 24–28 °C and 90 mm in five days on YES at 24–28 °C.		Insulated branched that forms filamentous hyphae	Conidiophores, globose vesicle, small round conidia, more formation on PDA than on YES		This study
*Aspergillus* sp, SMB-22	Cottony appearance, black in color. With formation of many black spores. The reverse is pale yellow and rough.	Less intense color, from white to black, with white concentric edges of more dispersed growth. Rough orange appearance on the reverse.		Several colonies grew at once, reaching 90 mm after the seventh day on PDA at 24–28 °C and 90 mm in five days on YES at 24–28 °C.		The hyphae are filamentous and branched.	Conidiophores, globose vesicle, small round conidia, black in color, more sporulation on PDA than on YES		This study
*Fusarium oxysporum*	White hairy mycelium, which turns purple after a week. No sporodochia formation. The reverse may be hyaline, dark blue or purple.			PDA: 30–55 mm. Colony diameter on PDA (Potato dextrose agar) after 4 days at 25 °C		Shape of chlamydosporess simple and double. Intercalary and terminal. Wall of medium or thin thickness.	Conidiogenous cells are monophyalides, microconidia are arranged in moist heads.		^61,62^
*Fusarium* sp, SMB-20	White aerial mycelium with a light purple base. Purple reverse with a white border.	White aerial mycelium with concentric growth. Pale orange reverse		Concentric growth reached 90 mm after the seventh day on PDA at 24–28 °C and 70 mm in five days on YES at 24–28 °C.		Insulated, branched, filamentous and septate hyphae ending in rounded conidiophores.	Elongated conidiophores with rounded ends. Typical oval, elliptic and kidney-shaped conidia.		This study
*Alternaria alternata*	Colonies effuse, usually grey, dark blackish brown or black. The reverse is typically brown to black due to pigment production.			*Alternaria* grow rapidly, the colony reaching a diameter of 3 to 9 cm after incubation at 25 °C for 7 days on PDA, greenish black or olive brown with a clear edge.		Mycelium immersed or partly superficial; hyphae colorless, olivaceous brown or brown. Stroma rarely formed. Setae and hyphopodia absent.	Conidiophores macronematous, mononematous, simple and loosely branched, pale brown or brown, in fascicles. Conidia catenate, dry, ovoid, pale or medium olivaceous-brown to brown, verrucose.		^37^
*Alternaria* sp. SMB-28	Colonies are slightly cottony, usually dark brown with thin white edges. The reverse is dark black.	Cottony colonies, usually dark brown with thick white edges. The reverse is orange-brown.		Individual colony growing concentrically, reaching 70 mm after nine days on PDA at 24–28 °C and 80 mm in seven days on YES at 24–28 °C.		Insulated, branched, filamentous and septate hyphae ending in rounded conidiophores.	Formation of few conidia on PDA and YES, pale brown spores, septate and warty.		This study

Molecular identification was conducted by amplifying the fungal 18S rDNA with the EF4f/EF3r primers, which successfully amplified the target sequences for all four fungal strains (SMB-18, SMB-20, SMB-22, and SMB-28). BLAST analysis of the resulting sequences identified these fungi as belonging to three distinct genera: *Fusarium* (SMB-20), *Aspergillus* (SMB-18, SMB-22), and *Alternaria* (SMB-28), confirming their classification as endophytic fungi (Figs. 2, 3, 4 and 5). Based on partial sequences of the 18S rDNA gene, phylogenetic analysis revealed that the isolated strains are closely related to other fungal species. However, species-level identification remained challenging due to the limited availability of comparable molecular data. The phylogenetic trees, shown in Figs. 2, 3, 4 and 5, were constructed using the Neighbor-Joining method, selecting the most suitable model based on sequence similarity. The fungal sequences obtained in this study were deposited in GenBank (Table 4). Given the low probability and the limited approach to the genus level, it is evident that an alternative locus is required for proper molecular identification and to expand the sequence data (Figs. 2, 3, 4 and 5).

### Morphological characteristics of endophytic fungi

The description of cultural and morphological characteristics of fungal endophytes, along with microphotographs of their morphological structures, is presented in Table 5; Fig. 6.

The morphological identification of the different strains of endophytic fungi in this study is primarily based on distinct characteristics, including the shape and size of macro- and microconidia, the presence or absence of chlamydospores, as well as colony appearance, pigmentation, and growth rate on agar media^45–47^.

Figure 6 presents the cultural and morphological characteristics of the studied endophytic fungi, including mycelial growth on PDA and YES, along with microphotographs of their hyphae, conidiophores, and conidia. Colonies of endophytic fungi typically exhibit rapid growth on PDA media at 30 °C, ranging between 3 and 7 days. However, growth is comparatively slower in YES culture media, taking between 7 and 10 days. Table 5 presents the macro- and microscopic characteristics of the species with strong similarities in comparison to the studied strains. Specifically, *Aspergillus niger* was used for comparison with *Aspergillus* sp. SMB-18 and SMB-22, *Fusarium oxysporum*, were compared with *Fusarium* sp. SMB-20 and *Alternaria alternata* were compared with *Alternaria* sp. SMB-28.

In *Aspergillus* species, the size and morphology of ascospores, particularly diagnostic features such as rugosity, margins, wings, and grooves, are essential for species identification^45,46^. *Aspergillus* strains SMB-18 and SMB-22 produce abundant black conidia on the surface and lack a cottony texture when grown on PDB (Fig. 6A, B). In contrast, sporulation was reduced on YES medium (Fig. 6C, D), where the strains exhibited improved growth, making them suitable for microscopic analysis. These strains form filamentous hyphae resembling miniature plants (Fig. 6E) (Table 5). Initially, the mycelium appeared white but turned black within two days on PDA, coinciding with conidial spore production. The reverse side of the colony displayed a light-yellow coloration. Notably, the conidiophores and conidia observed in both strains (Fig. 6E, F) are characteristic of the genus *Aspergillus* and closely resemble those of *Aspergillus niger*. *Fusarium* species were primarily identified based on distinctive characteristics, including the shape and size of macro- and microconidia, colony morphology, pigmentation, and growth rate on agar media^47^. The *Fusarium* sp. SMB-20 exhibited greater growth in PDA than in YES, showing different mycelial characteristics. On PDA, color development was more intense, pink-purple, and the texture appeared more cottony (Fig. 6A–D). Microscopic analysis revealed abundant filamentous hyphae on both PDA and YES, elongated conidiophores with rounded ends, and oval conidia, typical of the *Fusarium* genus (Fig. 6E, F, and Table 5). The genus *Alternaria* comprises primary conidiophores that can be straight or curved, varying in length from short to long, and may be either simple or branched, with one or multiple apical conidiophores^37^. In *Alternaria alternata*, the conidia are oval, elongated ellipsoid, small, and septate, resembling those observed in our study strain, *Alternaria* sp. SMB-28. This strain forms slow-growing colonies that produce few spores, yet these spores share key characteristics with *A. alternata*. Additionally, the similarity extends to other features, such as colony color on PDA (Fig. 6A-F; Table 5).

### Chemical profile of endophytic fungi extracts

The fungi *Fusarium* sp. SMB-20, *Aspergillus* sp. SMB-18, *Aspergillus* sp. SMB-22, and *Alternaria* sp. SMB-28 were cultivated in 200 mL of potato dextrose broth (PDB) for 15 days. Following cultivation, secondary metabolites were extracted from both the liquid media and fungal biomass using ethyl acetate. The yields of the extracts were 24.4 mg, 94.9 mg, 159.3 mg, and 36.1 mg for *Fusarium* sp. SMB-20, *Aspergillus* sp. SMB-18, *Aspergillus* sp. SMB-22, and *Alternaria* sp. SMB-28, respectively.

**Fig. 7:**
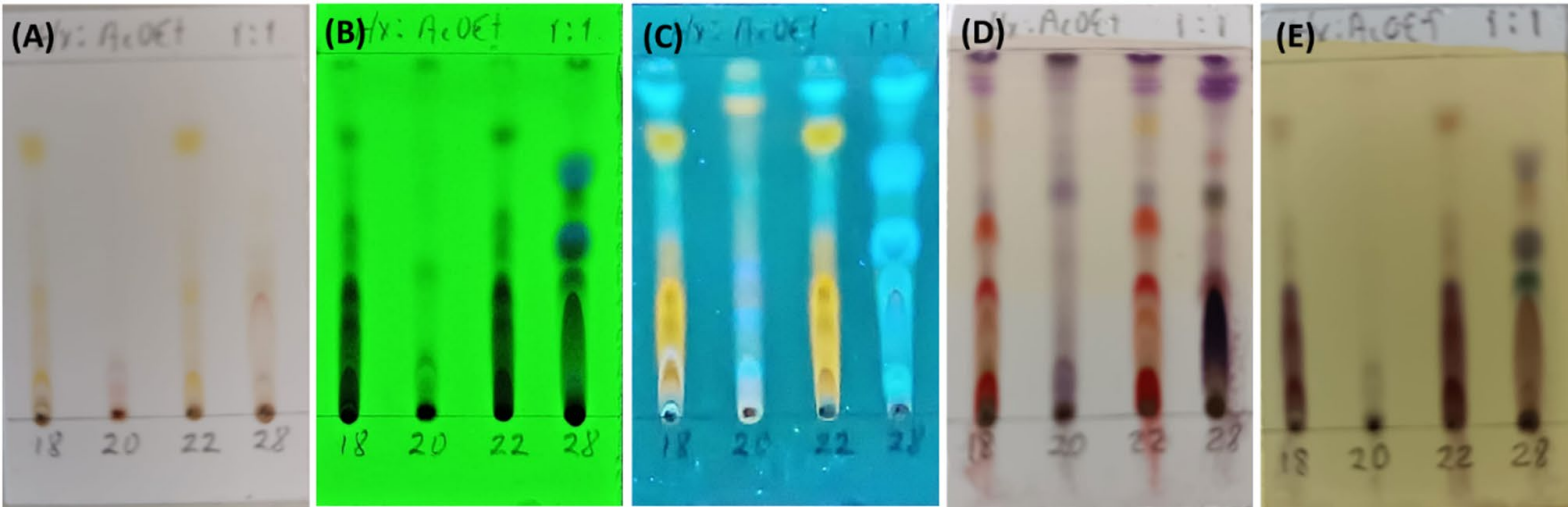
Thin-layer chromatography (TLC) of extracts from endophytic fungi strains, SMB-18, SMB-20, SMB-22, and SMB-28 that were shown in the TLC plate as 18, 20, 22, and 28, respectively. Compounds with chromophores or high unsaturation observed under visible light (A). The presence of conjugated double bonds observed under UV light at 254 nm (B). The presence of flavonoids stained with aluminum chloride and illuminated under UV light at 365 nm (C). Compounds stained with *p*-anisaldehyde, indicating the presence of terpenes (purple spots) and flavonoids (red spots) (D). Phenolic compounds stained with ferric chloride (E).

To identify the main groups of chemical compounds of the extracts, thin-layer chromatography (TLC) was employed, along with various staining techniques (Fig. 7A-E). The results from TLC analysis showed the presence of flavonoids, terpenoids, and phenolic compounds with visible chromophores under normal light for *Aspergillus* sp. SMB-18, SMB-22, and *Alternaria* sp. SMB-28 (Fig. 7A). However, no visible chromophores or highly unsaturated compounds were detected for *Fusarium* sp. SMB-20 under the same conditions.

Upon examination of the samples under UV light at 254 nm, conjugated double bonds were observed in all fungal extracts (Fig. 7B). Flavonoid compounds, which were stained with aluminum chloride and confirmed in all the extracts when exposed to UV light at 365 nm (Fig. 7C). Additionally, after developing the TLC plates with *p*-anisaldehyde, all four extracts exhibited purple spots with *Rf* values of 0.96 and 0.88, indicating the presence of terpenoid compounds. Red spots located at *Rf* values of 0.53 and 0.35 were identified in extracts from SMB-18 and SMB-22, suggesting the presence of flavonoids (Fig. 7D). These flavonoids were further corroborated by their appearance under UV light at 254 nm (Fig. 7B), confirming the presence of conjugated double bonds. Furthermore, the development of brown spots with ferric chloride suggested the presence of phenolic compounds, particularly in SMB-18, SMB-22, and SMB-28, with the latter showing a variety of phenolic compounds (Fig. 7E).

### Antimicrobial activity of crude Ethyl acetate extracts from endophytic fungi

The antimicrobial properties of crude extracts from endophytic fungi were evaluated against five bacterial strains: *Staphylococcus aureus*, *Escherichia coli*, *Pseudomonas aeruginosa*, *Enterococcus faecalis*, and *Propionibacterium acnes*. The minimum inhibitory concentrations (MICs) were determined for each ethyl acetate extract, providing insights into their efficacy against these pathogens.The findings showed that the extracts exhibited strong antibacterial activity against Gram-positive bacteria, with MICs ranging from 15.6 to 100 µg/mL, as shown in Tables 5 and 6. In contrast, no antimicrobial effect was observed against the Gram-negative strains, suggesting a selective antibacterial profile targeting Gram-positive bacteria. This differential activity suggests that the extracts could be specifically effective for applications targeting Gram-positive bacterial infections.

**Table 6:** Minimum inhibitory concentration (MIC) and Inhibition activity at various concentrations of crude extract prepared from endophytic fungi against 5 bacteria.

Endophytic Fungi	Bacteria	Inhibition (µg/mL)						
		1,000	500	250	125	62.5	31.3	15.6
*Aspergillus* sp. SMB-18	*S. aureus*	+++	++	++	++	+	-	-
	*E. coli*	-	-	-	-	-	-	-
	*P. aeruginosa*	-	-	-	-	-	-	-
	*E. faecalis*	+++	+	-	-	-	-	-
	*P. acnes*	+++	+++	+++	+++	++	++	++
*Fusarium* sp. SMB-20	*S. aureus*	+++	++	++	++	+	-	-
	*E. coli*	-	-	-	-	-	-	-
	*P. aeruginosa*	-	-	-	-	-	-	-
	*E. faecalis*	+++	+++	+++	+++	+++	+	-
	*P. acnes*	+++	+++	+++	+++	+++	++	-
*Aspergillus* sp. SMB-22	*S. aureus*	++	++	++	++	++	-	-
	*E. coli*	-	-	-	-	-	-	-
	*P. aeruginosa*	-	-	-	-	-	-	-
	*E. faecalis*	+++	+++	-	-	-	-	-
	*P. acnes*	+++	+++	++	++	++	++	++
*Alternaria* sp. SMB-28	*S. aureus*	++	++	++	-	-	-	-
	*E. coli*	-	-	-	-	-	-	-
	*P. aeruginosa*	-	-	-	-	-	-	-
	*E. faecalis*	++	++	+	-	-	-	-
	*P. acnes*	++	++	++	-	-	-	-

+++ : indicates the sample completely inhibited bacterial growth.

++ : indicates the sample completely inhibited bacterial growth by more than 90%.

+ : indicates the sample completely inhibited bacterial growth by more than 70%.

- : indicates no or less than 70% inhibition.

A closer look at the individual extracts revealed distinct patterns of inhibition. For instance, *Aspergillus* sp. SMB-18 exhibited strong antibacterial effects, where complete inhibition of *E. faecalis* was observed at a concentration of 1,000 µg/mL. It also significantly inhibited *S. aureus*, achieving substantial inhibition (++) down to 125 µg/mL. Notably, the extract demonstrated complete inhibition of *P. acnes* at 125 µg/mL and maintained strong inhibition down to 15.6 µg/mL, suggesting potential for use against *P. acnes*-related bacteria.

*Fusarium* sp. SMB-20 also demonstrated promising antibacterial properties. It completely inhibited *E. faecalis* and *P. acnes* at concentrations as low as 62.5 µg/mL, with substantial inhibition of *S. aureus* observed at 125 µg/mL. This indicates that *Fusarium* sp. SMB-20 may hold high promise in combating a range of Gram-positive bacterial pathogens.

The *Aspergillus* sp. SMB-22 extract was highly effective, particularly against *S. aureus* and *P. acnes*. A complete inhibition (+++) of *E. faecalis* and *P. acnes* was observed at 500 µg/mL. Moreover, substantial inhibition (++) of *S. aureus* was achieved at concentrations as low as 62.5 µg/mL. This suggests that *Aspergillus* sp. SMB-22 may be an excellent candidate for further exploration in the development of antibacterial agents. These results underscored the potential of *Aspergillus* sp. SMB-22 as a source of potent antimicrobial agents and highlighted the need for further investigation into the specific compounds responsible for this pronounced antibacterial activity.

*Alternaria* sp. SMB-28 also demonstrated antibacterial activity. The crude extract demonstrated inhibition of *S. aureus* across a range of concentrations, achieving complete inhibition by more than 90% (++) at 1,000 µg/mL, 500 µg/mL, and 250 µg/mL. The *Alternaria* sp. SMB-28 extract also showed antibacterial activity against *P. acnes* at 250 µg/mL, which further highlights its potential as a source for antibacterial agents.

**Table 7:** Summary of minimum inhibitory concentration (MIC) and Inhibition activity at 125 µg/mL of crude extract prepared from endophytic fungi.

Endophytic fungi	MIC (µg/mL)					Inhibition (%) at 125 µg/mL				
	S. Aureus	E. coli	*P*. aeruginosa	E. faecalis	*P*. acnes	S. Aureus	E. coli	*P*. aeruginosa	E. faecalis	*P*. acnes
*Aspergillus* sp. SMB-18	62.5	-	-	250	15.6	94.0 ± 1.68	-	-	32.6 ± 1.72	99.7 ± 0.18
*Fusarium* sp. SMB-20	250	-	-	31.3	31.3	34.9 ± 1.37	-	-	100 ± 0.08	99.6 ± 0.15
*Aspergillus* sp. SMB-22	62.5	-	-	500	15.6	97.7 ± 0.60	-	-	27.8 ± 2.29	99.6 ± 0.14
*Alternaria* sp. SMB-28	250	-	-	250	250	28.1 ± 1.00	-	-	11.0 ± 3.63	-

### Antifungal test: direct confrontation—dual culture plate assay

**Fig. 8:**
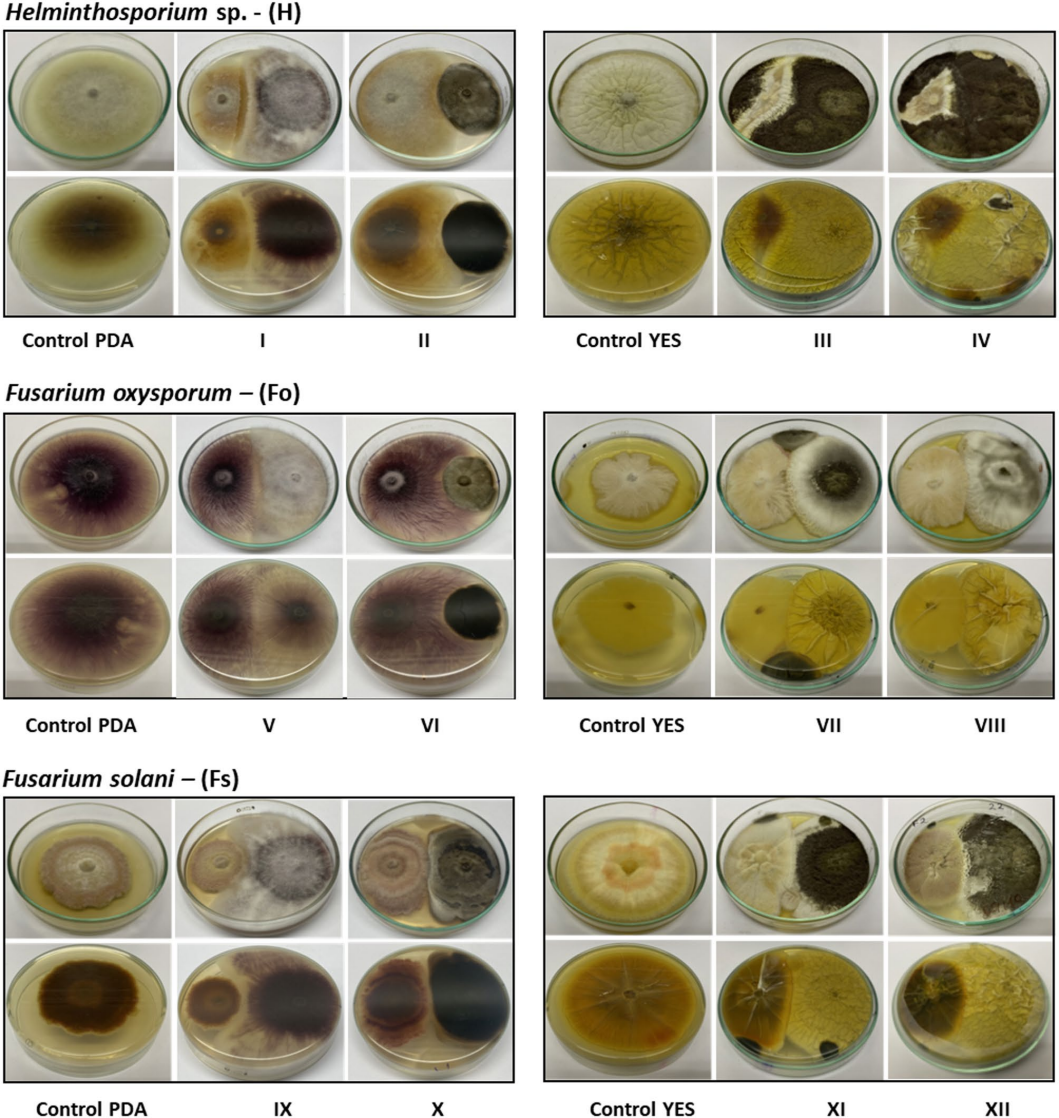
Direct confrontation of the endophytes *Aspergillus* sp. SMB-18, *Fusarium* sp. SMB-20, Aspergillus sp. SMB-22 and *Alternaria* sp. SMB-28 on the phytopathogens *Helminthosporium*, sp. (H), *Fusarium oxysporum* (Fo) and *Fusarium solani* (Fs). Confrontation of *Fusarium* sp. SMB-20 on PDA, on H (I), on Fo (V) and on Fs (IX). Confrontation of *Alternaria* sp. SMB-28 on PDA, on H (II), on Fo (VI) and on Fs (X). Confrontation of *Aspergillus* sp. SMB-18 on YES, on H (III), on Fo (VII) and on Fs (XI). Confrontation of *Aspergillus* sp. SMB-22 on YES, on H (IV), on Fo (VIII) and on Fs (XII).

The antagonistic potential of four endophytic fungi was evaluated through direct confrontation with phytopathogenic fungi using the dual culture plate assay. The inhibition of pathogen growth was quantified by measuring the radial mycelial extension in the confrontation assays (Fig. 8).

**Fig. 9:**
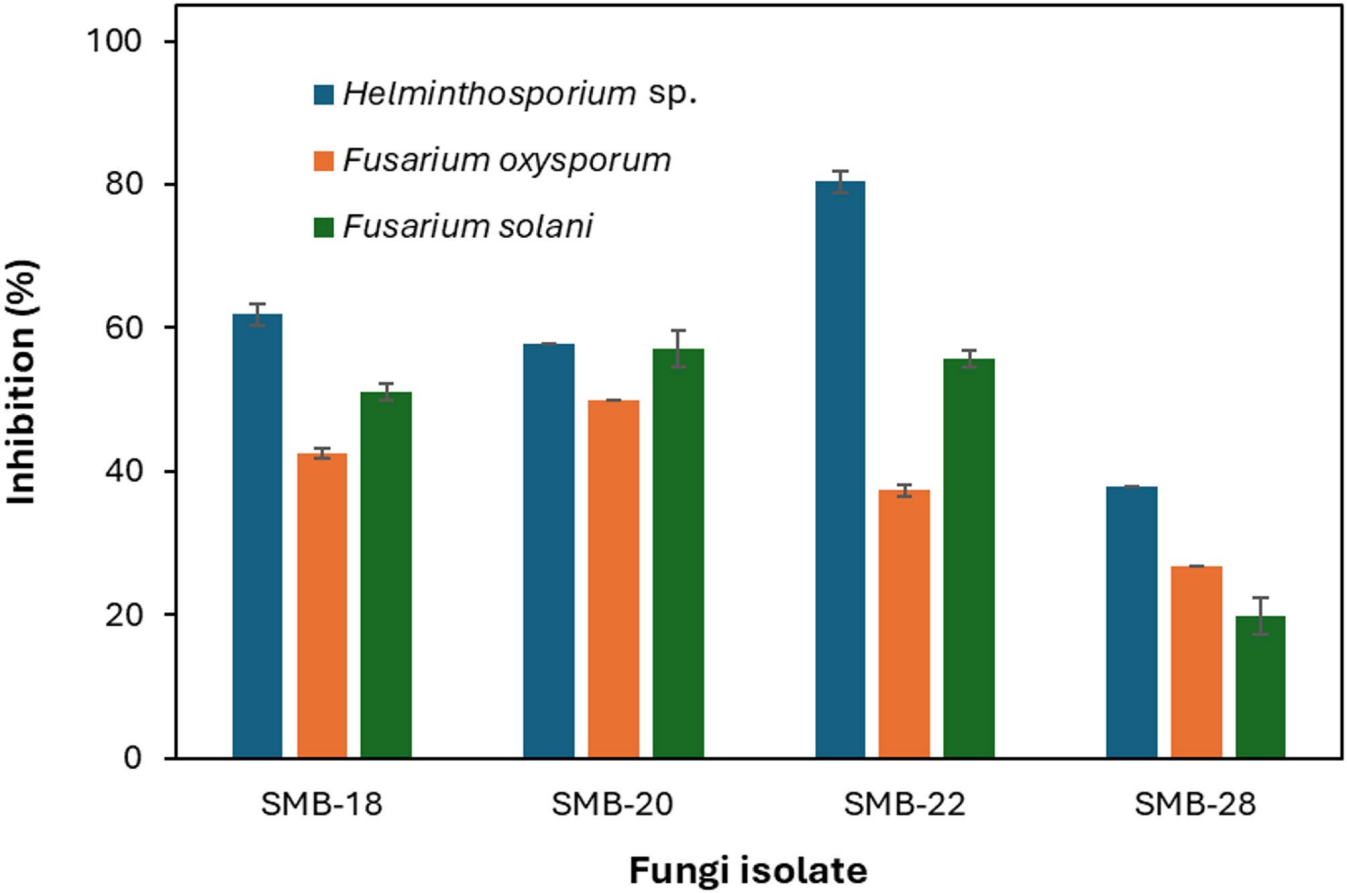
Percentage of inhibition of phytopathogen growth by endophytic fungi.

*Aspergillus* sp. SMB-18 exhibited substantial inhibition of *Helminthosporium* sp., *Fusarium oxysporum*, and *Fusarium solani* by 62%, 42%, and 51%, respectively. Similarly, *Fusarium* sp. SMB-20 also showed notable antagonistic effects, with inhibition rates of 58% against *Helminthosporium* sp., 50% against *F. oxysporum*, and 57% against *F. solani* (Fig. 9).

Among the tested isolates, *Aspergillus* sp. SMB-22 exhibited the highest inhibition against *Helminthosporium* sp., achieving an impressive 80% inhibition. It also inhibited *F. oxysporum* and *F. solani* by 37% and 56%, respectively. In contrast, *Alternaria* sp. SMB-28 demonstrated relatively lower antagonistic activity, with inhibition percentages of 38% for *Helminthosporium* sp., 27% for *F. oxysporum*, and 20% for *F. solani* (Fig. 9).

These results indicate that *Aspergillus* sp. SMB-18 and SMB-22 exhibit strong potential as biocontrol agents against phytopathogenic fungi, particularly *Helminthosporium* sp., while *Alternaria* sp. SMB-28 showed weaker inhibitory effects.

Based on the dual culture plate assay, six distinct types of interactions (A-F) (Table 3) were identified between the four endophytic fungi and three potato crop pathogens (Table 8): *Helminthosporium* sp., *Fusarium oxysporum*, and *Fusarium solani* (Fig. 8). All endophytes exhibited some level of inhibition against the pathogens, with no confrontations categorized as Type A (mutual growth without inhibition). Type B interactions were observed in the confrontations of *Fusarium* sp. SMB-20 with *F. oxysporum* (Fig. 8-Fo (V)), *Aspergillus* sp. SMB-22 with *F. oxysporum* (Fig. 8-Fo (VIII)), and *Alternaria* sp. SMB-28 with *F. solani* (Fig. 8-Fs (X)), where a minor inhibition zone was observed between the interacting fungi.

**Table 8:** Types of confrontation as a result of dual tests between endophytic and phytopathogenic fungi.

Phytophatogen	Aspergillus sp. SMB-18	Fusarium sp. SMB-20	Aspergillus sp. SMB-22	Alternaria sp. SMB-28
*Helminthosporium*, sp.	III – Type F	I– Type C	IV – Type F	II – Type D
*Fusarium oxysporum*	VII – Type E	V – Type B	VIII – Type B	VI – Type D
*Fusarium solani*	XI – Type E	IX – Type F	XII – Type E	X – Type B

Type C interactions, characterized by moderate inhibition, were noted between *Fusarium* sp. SMB-20 and *Helminthosporium* sp. (Fig. 8-H (I)), where the pathogen’s growth was slightly hindered in the presence of the endophyte. Type D interactions were recorded for *Alternaria* sp. SMB-28 against both *Helminthosporium* sp. (Fig. 8-H (II)) and *F. oxysporum* (Fig. 8-Fo (VI)), where inhibition zones of varying sizes were observed but not as prominent as in Type E or F interactions.

Types E and F represented the interactions with the highest inhibition levels of the pathogen. These interactions were predominantly observed in the *Aspergillus* endophytes, SMB-18 and SMB-22. Type E interactions, which displayed more substantial inhibition, were seen in the confrontations of *Aspergillus* sp. SMB-18 with *F. oxysporum* (Fig. 8-Fo (VII)), *Aspergillus* sp. SMB-18 with *F. solani* (Fig. 8-Fs (XI)), and *Aspergillus* sp. SMB-22 with *F. solani* (Fig. 8-Fs (XII)). Type F, which indicated the strongest inhibition, was observed in the interactions of *Aspergillus* sp. SMB-18 with *Helminthosporium* sp. (Fig. 8-H (III)) and *Aspergillus* sp. SMB-22 with *Helminthosporium* sp. (Fig. 8-H (IV)), where the pathogens showed considerable growth reduction.

The results from the interaction classifications highlight the varying degrees of antagonistic activity exhibited by the endophytes, with *Aspergillus* sp. strains SMB-18 and SMB-22 showing the most potent antagonistic effects against the potato crop pathogens.

## Discussion

The Amazon region of Bolivia is recognized as one of the most biodiverse areas in the world, as highlighted by Ibisch and Mérida^48^. Despite Bolivia’s biodiversity being underexplored, its immense natural wealth presents untapped potential for medicinal research. Nevertheless, the existing baseline data and their interpretation highlight the significance of Bolivia’s biodiversity at both national and international levels. This remarkable biodiversity underscores the region’s immense potential as a source of bioactive compounds for medicinal research. The deep connection between biodiversity and indigenous plant use creates a unique opportunity for discovering novel bioactive compounds from native plants, as well as their associated endophytic fungi. These microorganisms are known to produce a wide variety of bioactive metabolites that can be explored for promising applications in agriculture, medicine, and biotechnology. In this context, the traditional use of the plants and their ecological habitats are crucial factors to consider when isolating endophytes.

Our study, which focused on the isolation of endophytic fungi from plants in the Bolivian Amazon, aligns with the idea that regions with high biodiversity as likely to harbor unique microbial communities. Four fungal isolates, *Aspergillus* sp. SMB-18, *Aspergillus* sp. SMB-22, *Fusarium* sp. SMB-20, and *Alternaria* sp. SMB-28, were successfully isolated from the leaves of medicinal plants (*Piper heterophyllum* Ruiz & Pav., *Peperomia* sp., *Faramea multiflora* A. Rich. Ex DC., and *Dictyoloma vandellianum* A. Juss.). These results are consistent with previous studies emphasizing the rich phylogenetic diversity of endophytic fungi in tropical ecosystems, driven by the high diversity of host plants and environmental factors^14,49^.

Notably, while *Aspergillus* sp. SMB-18 and SMB-22 belong to the same genus; they exhibited distinct antagonistic profiles, supporting the hypothesis that even closely related species may evolve different adaptations based on their host environments. The isolation of *Fusarium* and *Alternaria* strains further supports the growing evidence of these genera being prevalent in tropical endophytic fungal communities^50,51^.

The molecular identification and phylogenetic analysis of these fungi demonstrate the need for improved species-level resolution. As we observed, utilizing a single genetic marker for identification may not be sufficient, particularly when distinguishing between closely related fungal species^35,52^. Chemical profiling of the fungal extracts revealed a wide array of secondary metabolites, such as flavonoids, terpenes, and phenolic compounds. The identification of these metabolites through TLC provides strong evidence for the biochemical potential of these endophytic fungi as sources of bioactive compounds^5,15^. Flavonoids and terpenes are well-known for their antimicrobial, antioxidant, and anti-inflammatory properties, which may explain the observed antimicrobial activities of the fungal extracts^5^. The presence of these compounds, particularly flavonoids with conjugated double bonds and terpenes observed under UV light, aligns with the antimicrobial activities demonstrated by these extracts. Moreover, the phenolic compounds detected, which are known for their ability to disrupt bacterial cell membranes, further support the idea that these extracts could have therapeutic value, particularly in antibacterial applications^53^.

The antimicrobial activity of the crude fungal extracts demonstrated selective efficacy against Gram-positive bacteria, including *S. aureus*, *E. faecalis*, and *P. acnes* (Table 6). This selective activity is likely due to the simpler cell wall structure of Gram-positive bacteria compared to Gram-negative bacteria, which may allow for better penetration and inhibition by the bioactive compounds^54–56^. The observed MICs ranged from 15.6 to 500 µg/mL. *Aspergillus* sp. SMB-18 and SMB-22 exhibited particularly strong activity against *S. aureus* and *E. faecalis*, with inhibition percentages reaching as high as 97.7% at 125 µg/mL. Although the MICs of the fungal extracts were less potent than the positive control, ampicillin, these results suggest that the fungi have considerable antibacterial properties, especially at higher concentrations. *Fusarium* sp. SMB-20 exhibited noteworthy activity with a MIC of 31.3 µg/mL to *E. faecalis*, making it a particularly promising candidate for future antibacterial research. Against *P. acnes*, a Gram-positive bacterium of dermatological relevance, *Aspergillus* sp. SMB-18, *Fusarium* sp. SMB-20, and *Aspergillus* sp. SMB-22 achieved 100% inhibition at 125 µg/mL. This highlights the potential of these fungi in addressing skin-related bacterial infections^57^. These findings demonstrate that certain endophytic fungi, particularly *Aspergillus* and *Fusarium* species, possess strong antibacterial properties. Future studies should focus on isolating and characterizing the specific bioactive metabolites responsible for these activities, which could lead to the development of novel antibiotics for clinical use.

The dual culture plate assay, used to assess the antifungal properties of the endophytes, revealed diverse antagonistic interactions between the fungi and three potato crop pathogens (*Helminthosporium* sp., *Fusarium oxysporum*, and *Fusarium solani*). Five distinct interaction types (B-F) were identified, reflecting the range of inhibitory responses exhibited by the fungi. Type E interactions, which showed the most strong inhibition, were observed in confrontations between *Aspergillus* sp. SMB-18 and both *F. oxysporum* and *F. solani*, as well as between *Aspergillus* sp. SMB-22 and *F. solani*. These results highlight the potential of these fungi, particularly *Aspergillus* species, as biocontrol agents for managing phytopathogenic fungi. Additionally, *Fusarium* sp. SMB-20 demonstrated Type C and B interactions, indicating its versatility in targeting both *Helminthosporium* sp. and *F. oxysporum*. The lower inhibition observed with *Alternaria* sp. SMB-28 suggests that its antifungal potential may be less pronounced, though it still exhibited some activity against *F. solani*.

Overall, the diverse interaction types and the promising antimicrobial activities demonstrated by these endophytic fungi suggest that they could serve as valuable sources of secondary metabolites with potential applications in medicine, agriculture, and biocontrol.

## Conclusions

In this study, the isolation and identification of four endophytic fungi, *Fusarium* sp. SMB-20, *Aspergillus* sp. SMB-18 and SMB-22, and *Alternaria* sp. SMB-28, were successfully achieved from the leaves of different Amazonian medicinal plants in Bolivia: *Piper heterophyllum* Ruiz & Pav., *Peperomia* sp., *Faramea multiflora* A. Rich. ex DC., and *Dictyoloma vandellianum* A. Juss. The crude extracts from these fungi, cultured in PDB, demonstrated strong antibacterial activity against *S. aureus*, *E. faecalis*, and *P. acnes*, with MIC values ranging from 15.6 to 500 µg/mL. This selective efficacy against Gram-positive bacteria highlights the potential of these fungi as sources of bioactive compounds with antimicrobial properties. In antifungal assays, the dual culture plate method revealed varying degrees of antagonistic interactions between the isolated endophytes and the phytopathogens *Helminthosporium* sp., *Fusarium oxysporum*, and *Fusarium solani*. Notably, *Aspergillus* sp. SMB-18 and SMB-22 exhibited the highest inhibitory effects, classified under interaction types E and F, demonstrating strong potential as biocontrol agents against plant pathogens.

These findings underscore the significance of endophytic fungi as promising reservoirs of biologically active secondary metabolites with potential applications in medicine, pharmaceuticals, agriculture, and environmental biotechnology. Further studies should focus on the isolation, structural characterization, and mode of action of these bioactive compounds to harness their full potential for therapeutic and agricultural innovations.

## Data Availability

All authors declare that all data generated or analysed during this study are included in this manuscript.

## References

[1] Guo, B., Wang, Y., Sun, X. & Tang, K. Bioactive natural products from endophytes: a review. *Prikl Biokhim. Mikrobiol*. **44** (2), 153–158 (2008). 18669256

[2] Cruz, J. S., da Silva, C. A. & Hamerski, L. Natural products from endophytic fungi associated with Rubiaceae species. **6**(3), 128 (2020). 10.3390/jof6030128 PMC7558492 32784526

[3] Hercegová - Firáková, S., Sturdikova, M. & Múčková, M. Bioactive secondary metabolites produced by microorganisms associated with plants. *Biologia* **62**, 251–257. 10.2478/s11756-007-0044-1 (2007).

[4] Jia, M. et al. A friendly relationship between endophytic fungi and medicinal plants: A systematic review. 10.3389/fmicb.2016.00906 (2016). 10.3389/fmicb.2016.00906 PMC4899461 27375610

[5] Gakuubi, M. M., Munusamy, M., Liang, Z. X. & Ng, S. B. Fungal endophytes: A promising frontier for discovery of novel bioactive compounds. *J. Fungi (Basel Switzerland)*. **7** (10). 10.3390/jof7100786 (2021). 10.3390/jof7100786 PMC8538612 34682208

[6] Cieniecka-Rosłonkiewicz, A. M. A. & Cholewińska, M. Plant endophytic fungi as a source of Paclitaxel. *Herba Pol.* **60** (4). 10.1515/hepo-2015-0002 (2014).

[7] Stierle, A., Strobel, G. & Stierle, D. Taxol and taxane production by taxomyces andreanae, an endophytic fungus of Pacific Yew. *Science* **260** (5105), 214–216. 10.1126/science.8097061 (1993). 8097061 10.1126/science.8097061

[8] Devari, S. et al. Capsaicin production by alternaria alternata, an endophytic fungus from capsicum annum; LC-ESI-MS/MS analysis. *Phytochemistry* **98**, 183–189. 10.1016/j.phytochem.2013.12.001 (2014). 24378219 10.1016/j.phytochem.2013.12.001

[9] Chithra, S., Jasim, B., Sachidanandan, P., Jyothis, M. & Radhakrishnan, E. K. Piperine production by endophytic fungus Colletotrichum gloeosporioides isolated from Piper nigrum. *Phytomedicine: Int. J. Phytotherapy Phytopharmacology*. **21** (4), 534–540. 10.1016/j.phymed.2013.10.020 (2014). 10.1016/j.phymed.2013.10.020 24268806

[10] Alvin, A., Miller, K. I. & Neilan, B. A. Exploring the potential of endophytes from medicinal plants as sources of antimycobacterial compounds. *Microbiol. Res.* **169** (7–8), 483–495. 10.1016/j.micres.2013.12.009 (2014). 24582778 10.1016/j.micres.2013.12.009 PMC7126926

[11] Gunatilaka, A. A. Natural products from plant-associated microorganisms: distribution, structural diversity, bioactivity, and implications of their occurrence. *J. Nat. Prod.* **69** (3), 509–526. 10.1021/np058128n (2006). 16562864 10.1021/np058128n PMC3362121

[12] Fadiji, A. E. & Babalola, O. O. Elucidating mechanisms of endophytes used in plant protection and other bioactivities with multifunctional prospects. *Front. Bioeng. Biotechnol.*10.3389/fbioe.2020.00467 (2020). 32500068 10.3389/fbioe.2020.00467 PMC7242734

[13] Strobel, G. The emergence of endophytic microbes and their biological promise. *J. Fungi (Basel Switzerland)*. **4** (2). 10.3390/jof4020057 (2018). 10.3390/jof4020057 PMC6023353 29772685

[14] Strobel, G. & Daisy, B. Bioprospecting for microbial endophytes and their natural products. *Microbiol. Mol. Biol. Rev.* **67** (4), 491–502. 10.1128/mmbr.67.4.491-502.2003 (2003). 14665674 10.1128/MMBR.67.4.491-502.2003 PMC309047

[15] Zhang, H. W., Song, Y. C. & Tan, R. X. Biology and chemistry of endophytes. *Nat. Prod. Rep.* **23** (5), 753–771. 10.1039/b609472b (2006). 17003908 10.1039/b609472b

[16] Deshmukh, S. K., Gupta, M. K., Prakash, V. & Saxena, S. Endophytic fungi: A source of potential antifungal compounds. *J. Fungi (Basel)*. **4** (3). 10.3390/jof4030077 (2018). 10.3390/jof4030077 PMC6162562 29941838

[17] Aly, A. H., Debbab, A. & Proksch, P. Fungal endophytes: unique plant inhabitants with great promises. *Appl. Microbiol. Biotechnol.* **90** (6), 1829–1845. 10.1007/s00253-011-3270-y (2011). 21523479 10.1007/s00253-011-3270-y

[18] Gouda, S., Das, G., Sen, S. K., Shin, H.-S. & Patra, J. K. Endophytes: A treasure house of bioactive compounds of medicinal importance. *Front. Microbiol.*10.3389/fmicb.2016.01538 (2016). 27746767 10.3389/fmicb.2016.01538 PMC5041141

[19] Vedamurthy, A. B., Mane, R. S. & Paarakh, P. M. Brief review on fungal endophytes. *Int. J. Secondary Metabolite*. **5** (4), 288–303. 10.21448/ijsm.482798 (2018).

[20] Ghareeb, R. Y. et al. Biocontrol potential of endophytic fungi against phytopathogenic nematodes on potato (Solanum tuberosum L). *Sci. Rep.* **14** (1), 15547. 10.1038/s41598-024-64056-x (2024). 38969662 10.1038/s41598-024-64056-x PMC11229511

[21] PROINPA & Fundación Promoción e Investigación de Productos Andinos. (accessed 02 Feb 2024). https://www.proinpa.org/web/.

[22] Salehi, B. et al. Piper species: A comprehensive review on their phytochemistry. *Biol. Activities Appl.* **24** (7), 1364 (2019). 10.3390/molecules24071364 PMC6479398 30959974

[23] Teodhora, Hendriani, R., Sumiwi, S. A. & Levita, J. Peperomia pellucida (L.) kunth: A decade of Ethnopharmacological, Phytochemical, and Pharmacological insights (2014–2025). *J. Experimental Pharmacol.* **17** (null), 417–454. 10.2147/JEP.S532898 (2025). 10.2147/JEP.S532898 PMC12235017 40630672

[24] Delprete, P. & Jardim, J. Systematics, taxonomy and floristics of Brazilian rubiaceae: an overview about the current status and future challenges. *Rodriguésia* **63**, 101–128. 10.1590/S2175-78602012000100009 (2012).

[25] Sartor, C. F. et al. Alkaloids from Dictyoloma vandellianum: their chemosystematic significance. *Phytochemistry* **63** (2), 185–192. 10.1016/s0031-9422(03)00006-2 (2003). 12711140 10.1016/s0031-9422(03)00006-2

[26] Girault, L. Kallawaya curanderos itinerantes de los Andes: investigación sobre práticas medicinales y mágicas. Quipus (1987).

[27] Macía, M., García, E. & Vidaurre, P. An ethnobotanical survey of medicinal plants commercialized in the markets of La Paz and El Alto. *Bolivia J. Ethnopharmacol.* **97**, 337–350. 10.1016/j.jep.2004.11.022 (2005). 15707774 10.1016/j.jep.2004.11.022

[28] Griffith, G. W. et al. Copper deficiency in potato dextrose agar causes reduced pigmentation in cultures of various fungi. *FEMS Microbiol. Lett.* **276** (2), 165–171. 10.1111/j.1574-6968.2007.00923.x (2007). 17956422 10.1111/j.1574-6968.2007.00923.x

[29] Schulz, B. & Boyle, C. The endophytic continuum. *Mycol. Res.* **109** (6), 661–686. 10.1017/S095375620500273X (2005). 16080390 10.1017/s095375620500273x

[30] Tandon, T., Jain, P. & Kumar, T. Isolation and identification of endophytic fungi from Indigenous medicinal plants. *Biosci. Biotechnol. Res. Asia*. **22**, 201–208. 10.13005/bbra/3354 (2025).

[31] Araújo WLd, Lima, A. O. S., Azevedo JLd, Marcon, J., Sobral, J. K. & Lacava, P. T. Manual: isolamento de microrganismos endofiticos.

[32] Mendieta-Brito, S. et al. Identification, Characterization, and antibacterial evaluation of five endophytic fungi from psychotria Poeppigiana Müll. *Arg Amazon Plant.* **12** (8), 1590 (2024). 39203432 10.3390/microorganisms12081590 PMC11356722

[33] Stinson, M., Ezra, D., Hess, W. M., Sears, J. & Strobel, G. An endophytic gliocladium sp. of eucryphia cordifolia producing selective volatile antimicrobial compounds. *Plant Sci.* **165** (4), 913–922. 10.1016/S0168-9452(03)00299-1 (2003).

[34] Chen, Y-J., Chen, H-J. & Chung, W-H. Endophytic fungal diversity in *Cirsium kawakamii* from Taiwan. **9**(11):1076 (2023). 10.3390/jof9111076 PMC10671896 37998881

[35] Samson, R. A. et al. Phylogeny, identification and nomenclature of the genus Aspergillus. *Stud. Mycol.* **78**, 141–173. 10.1016/j.simyco.2014.07.004 (2014). 25492982 10.1016/j.simyco.2014.07.004 PMC4260807

[36] Watanabe, T. Pictorial Atlas of Soil and Seed Fungi: Morphologies of Cultured Fungi and Key to Species, Second Edition (2002).

[37] Woudenberg, J. H., Groenewald, J. Z., Binder, M. & Crous, P. W. Alternaria redefined. *Stud. Mycol.* **75** (1), 171–212. 10.3114/sim0015 (2013). 24014900 10.3114/sim0015 PMC3713888

[38] Research, Z. Quick-DNA™ Fungal/Bacterial Miniprep Kit [Available from: https://zymoresearch.com/. [Accessed: 02–02, 2024].

[39] Gurgel, R. S. et al. Antimicrobial and antioxidant activities of endophytic fungi associated with arrabidaea Chica (Bignoniaceae). *J. Fungi (Basel Switzerland)*. **9** (8). 10.3390/jof9080864 (2023). 10.3390/jof9080864 PMC10455555 37623634

[40] CLSI & Standard CLSI M38. Reference Method for Broth Dilution Antifungal Susceptibility Testing of Filamentous Fungi [Wayne, PA, USA:[Available from: https://clsi.org/. [Accessed: 05–06, 2022].

[41] Dalsgaard, I. Selection of media for antimicrobial susceptibility testing of fish pathogenic bacteria. *Aquaculture* **196**, 267–275. 10.1016/S0044-8486(01)00538-5 (2001).

[42] Rigerte, L., Blumenstein, K. & Terhonen, E. New R-Based methodology to optimize the identification of root endophytes against heterobasidion parviporum. *Microorganisms* **7** (4), 102. 10.3390/microorganisms7040102 (2019). 30959873 10.3390/microorganisms7040102 PMC6517935

[43] Matarese, F., Sarrocco, S., Gruber, S., Seidl-Seiboth, V. & Vannacci, G. Biocontrol of fusarium head blight: interactions between trichoderma and mycotoxigenic fusarium. *Microbiol. (Reading England)*. **158** (Pt 1), 98–106. 10.1099/mic.0.052639-0 (2012). 10.1099/mic.0.052639-0 21980117

[44] Holkar, S. K. et al. Biocontrol potential of endophytic fungi originated from grapevine leaves for management of anthracnose disease caused by Colletotrichum gloeosporioides. *3 Biotech.* **13** (7), 258. 10.1007/s13205-023-03675-z (2023). 37405269 10.1007/s13205-023-03675-z PMC10314888

[45] Zulkifli, N. A. & Zakaria, L. Morphological and molecular diversity of Aspergillus from corn grain used as livestock feed. *HAYATI J. Biosci.* **24** (1), 26–34. 10.1016/j.hjb.2017.05.002 (2017).

[46] Okuda, T., Klich, M., Seifert, K. & Ando, K. *Media and Incubation Effects on Morphological Characteristics of Penicillium and Aspergillus* (Harwood Academic, 2000).

[47] Leslie JFaS, B. A. *The Fusarium laboratory manual* 1st edn. (Blackwell Publishing, 2006).

[48] Ibisch, P., Mérida, G. & Biodiversity *The Richness of Bolivia. State of Knowledge and Conservation* 1st edn (Santa Cruz de la Sierra, 2004).

[49] Schulz, B., Boyle, C., Draeger, S., Römmert, A-K. & Krohn, K. Endophytic fungi: a source of novel biologically active secondary metabolites* *Paper presented at the British mycological society symposium on fungal bioactive Compounds, held at the university of Wales Swansea on 22–27 April 2001. *Mycol. Res.* **106** (9), 996–1004. 10.1017/S0953756202006342 (2002).

[50] De Melo Wanderley Costa, P. et al. Checklist of endophytic fungi from tropical regions. *Mycotaxon*10.5248/119.493 (2012).

[51] Ting, A. S. Y. *Endophytes of the Tropics: Diversity, Ubiquity and Applications* 1st edn. (CRC, 2020).

[52] Adeyemo, A. & Schmidt-Heydt, M. Expansion of the multi-locus gene alignment approach to improve identification of the fungal species alternaria alternata. *Int. J. Food Microbiol.* **421**, 110746. 10.1016/j.ijfoodmicro.2024.110746 (2024). 38917488 10.1016/j.ijfoodmicro.2024.110746

[53] Lima, M. C. et al. A review of the current evidence of fruit phenolic compounds as potential antimicrobials against pathogenic bacteria. *Microb. Pathog*. **130**, 259–270. 10.1016/j.micpath.2019.03.025 (2019). 30917922 10.1016/j.micpath.2019.03.025

[54] Ghazi-Yaker, A. et al. In vitro antioxidant and antibacterial activities of Ethyl acetate extracts of Ziziphus Lotus leaves and five associated endophytic fungi. *Microorganisms* **12** (12), 2671 (2024). 39770873 10.3390/microorganisms12122671 PMC11728511

[55] Garcia, A. V. F. et al. Antimicrobial activity of crude extracts of endophytic fungi isolated from medicinal plant Sapindus saponaria L. *J. Appl. Pharm. Sci.* **2**, 35–40. 10.7324/JAPS.2012.21007 (2012).

[56] Hoque, N. et al. Antimicrobial, antioxidant, And cytotoxic properties of endophytic fungi isolated from Thysanolaena maxima Roxb., Dracaena spicata Roxb. And Aglaonema hookerianum Schott. *BMC Complement. Med. Ther.* **23** (1), 347. 10.1186/s12906-023-04185-4 (2023). 37777711 10.1186/s12906-023-04185-4 PMC10542267

[57] Deshmukh, S. K., Verekar, S. A. & Bhave, S. V. Endophytic fungi: a reservoir of antibacterials. *Front. Microbiol.* **5**, 715. 10.3389/fmicb.2014.00715 (2014). 25620957 10.3389/fmicb.2014.00715 PMC4288058

[58] Huang, W-Y. et al. Molecular phylogenetic identification of endophytic fungi isolated from three Artemisia species. *Fungal Divers.* **36**, 69–88 (2009).

[59] Selim, K. A., El-Beih, A. A., Abdel-Rahman, T. M. & El-Diwany, A. I. Biological evaluation of endophytic fungus, Chaetomium globosum JN711454, as potential candidate for improving drug discovery. *Cell Biochem. Biophys.* **68** (1), 67–82. 10.1007/s12013-013-9695-4 (2014). 23775636 10.1007/s12013-013-9695-4

[60] van Elsas, J. D., Duarte, G. F., Keijzer-Wolters, A. & Smit, E. Analysis of the dynamics of fungal communities in soil via fungal-specific PCR of soil DNA followed by denaturing gradient gel electrophoresis. *J. Microbiol. Methods*. **43** (2), 133–151. 10.1016/s0167-7012(00)00212-8 (2000). 11121612 10.1016/s0167-7012(00)00212-8

[61] Hafizi, R., Salleh, B. & Latiffah, Z. Morphological and molecular characterization of Fusarium. Solani and F. oxysporum associated with crown disease of oil palm. *Brazilian J. Microbiology: [publication Brazilian Soc. Microbiology]*. **44** (3), 959–968. 10.1590/s1517-83822013000300047 (2013). 10.1590/s1517-83822013000300047 PMC3910218 24516465

[62] Batt, C. A. & Tortorello, M. L. Encyclopedia of Food Microbiology, Second Edition, 1-2907 (2014).

